# Preconception care: delivery strategies and packages for care

**DOI:** 10.1186/1742-4755-11-S3-S7

**Published:** 2014-09-26

**Authors:** Zohra S Lassi, Sohni V Dean, Dania Mallick, Zulfiqar A Bhutta

**Affiliations:** 1 Division of Women and Child Health, Aga Khan University Karachi, Pakistan

**Keywords:** preconception, packages of care, continuum of care

## Abstract

The notion of preconception care aims to target the existing risks before pregnancy, whereby resources may be used to improve reproductive health and optimize knowledge before conceiving. The preconception period provides an opportunity to intervene earlier to optimize the health of potential mothers (and fathers) and to prevent harmful exposures from affecting the developing fetus. These interventions include birth spacing and preventing teenage pregnancy, promotion of contraceptive use, optimization of weight and micronutrient status, prevention and management of infectious diseases, and screening for and managing chronic conditions. Given existing interventions and the need to organize services to optimize delivery of care in a logical and effective manner, interventions are frequently co-packaged or bundled together. This paper highlights packages of preconception interventions that can be combined and co-delivered to women through various delivery channels and provides a logical framework for development of such packages in varying contexts.

## Introduction

Given the extent of the burden of maternal, newborn and child mortality; maternal, newborn and child health (MNCH) interventions can be grouped together into package as part of continuum of care. This approach includes integrated service delivery for mothers and children from pregnancy to delivery, extending into the immediate postnatal period, and childhood. The MNCH continuum of care approach was introduced on the grounds that the health and well-being of women, newborns, and children are closely linked and should be managed in a unified way [1]. Moreover, delivering interventions in the form of packages promote greater efficiency by maximizing synergies and avoiding duplication of services compared to when delivered alone [2]. However, even with increased prenatal and postnatal care practices, there was a slow improvement in birth outcomes and this led to the realization that prenatal care might be a step too late for pregnant mothers. Thus, the targeted MNCH momentum was expanded to include reproductive health as an essential component. The recent Lancet neonatal series reviewed 39 preconception and antenatal interventions, of which only two were targeted interventions for the preconception period [3]. The review further suggested six packages along the continuum of care for 75 countdown countries of which only one package included interventions for preconception period [3]. The package included interventions directed at improving nutritional status through balanced energy protein supplementation, folic acid supplementation/fortification, and micronutrient supplementation. Correcting nutritional status before pregnancy is important but changing the dynamics of maternal, neonatal and child health indices require holistic approach. Interventions to prevent and treat infection, chronic diseases, mental health and awareness of reproductive health are crucial elements and should also be targeted before pregnancy for ensuring improved pregnancy, neonatal and child health outcomes.

Therefore, the notion of preconception care aims to target the existing risks before pregnancy, whereby resources may be used to improve reproductive health of women, men and couples in order to optimize health and knowledge before conceiving a pregnancy. More recently, greater attention is being paid to the period before pregnancy, specifically focusing on preconception care. The preconception period provides an opportunity to intervene earlier to optimize the health of potential mothers (and fathers) and to prevent harmful exposures from affecting the developing foetus. These interventions include birth spacing and prevention of teenage pregnancy as young mothers often are not physically mature enough to deliver a baby, leaving them and their children at risk for death or disability from obstructed labor, fistulas, premature birth, or low birth weight. At the same time, early childbearing negatively affects educational and economic opportunities; women with lower educational attainment have greater risks of adverse pregnancy outcomes, are less knowledgeable about health-prevention activities, and family planning. Their children have fewer options for education, optimal growth and development and have a higher risk of mortality.

Preconception care is the provision of biomedical, behavioral and social health interventions to women and couples before conception occurs, aimed at improving their health status, and reducing behaviours and individual and environmental factors that could contribute to poor maternal and child health outcomes [4]. Its ultimate aim is improved maternal and child health outcomes, in both the short and long term. For the purpose of this review, ***preconception care*** and its boundaries were defined as: “any preventive, promotive or curative health care intervention provided to women of childbearing age in the perio*d* before pregnancy (at least 2 years) or between consecutive pregnancies, to improve health related outcomes for women (regardless of their pregnancy status), newborns or children up to 5 years of age” [5].

This paper summarizes key preconception risks and interventions which impact maternal, fetal, neonatal and child health outcomes (Refer to Paper 1-6), and proposes packages of evidence-based effective interventions for improved reproductive health and pregnancy outcomes. The paper also proposes strategies for delivery for such packages in varying contexts.

### Proposed packages of care

We identified and recommended various interventions through systematic reviews [6-11] and evidence synthesis that are summarized in Table 1 according to the level of care at which each can be provided.

**Table 1:** Preconception interventions and their delivery according to the level of care

Interventions	Level of care		
	
	Community	Primary	Referral
**Promoting Reproductive health**			

Promoting adolescent health	✔	✔	

Preventing first and repeat pregnancy in adolescence	✔	✔	

Birth spacing	✔	✔	✔

Reproductive planning after abortion			✔

Advanced maternal age	✔	✔	

Genetic counselling		✔	✔

**Nutritional status and supplementation**			

Maternal pre-pregnancy weight	✔	✔	

Diet, exercise and weight loss	✔	✔	✔

Folic acid supplementation	✔	✔	

Multivitamins supplementation	✔	✔	

Iron supplementation	✔	✔	

**Preventing and treating infections**			

Sexually transmitted infections	✔	✔	✔

HIV/AIDS prevention strategies	✔	✔	✔

Vaccine usage	✔	✔	✔

Periodontal disease and dental caries		✔	✔

Cytomegalovirus		✔	✔

**Screening and management of chronic diseases**			

Diabetes		✔	✔

Epilepsy management			✔

Management of Phenylketonuria			✔

Thyroid disorders			✔

Systemic Lupus Erythromatoses and other connective tissue diseases			✔

Medication use		✔	✔

Mental health	✔	✔	

Intimate partner violence	✔	✔	

**Substance abuse and lifestyle changes**			

Caffeine intake	✔	✔	

Alcohol intake	✔	✔	

Smoking cessation	✔	✔	

Illicit drugs consumption	✔	✔	

Ameliorating environmental exposures such as chemical and radiations	✔	✔	

**Figure 1:**
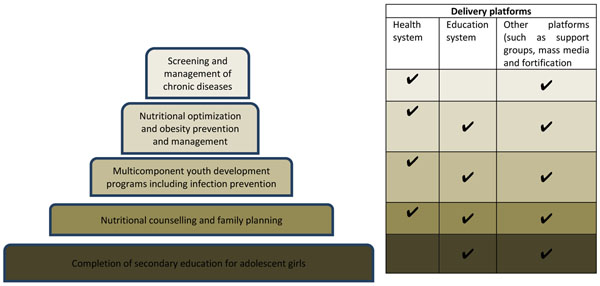
Different packages of preconception care interventions

These interventions can also be bundled together and delivered in various packages to increase efficiency and give a synergistic effect. Figure 1 illustrates different packages that can be delivered through various delivery platforms. It is important to highlight that individual interventions included in these packages can be modified according to local context, priorities as well as feasibility of delivery within particular levels of the health and education systems. To illustrate, these interventions can be bundled into the following five packages for delivery.

1. Completion of secondary education for adolescent girls and prevention of teenage pregnancy

2. Nutritional counselling and family planning

3. Nutritional optimization and weight loss programs

4. Multicomponent youth development programs including infection prevention

5. Screening and management of chronic diseases including mental health

#### 1. Completion of secondary education for adolescent girls and prevention of teenage pregnancy

To prevent teenage pregnancy and improve preconception care this package proposes comprehensive counselling, education and life skills development. Published papers highlighted the evidence regarding the adverse effects of teenage pregnancy on maternal and neonatal outcomes [12-15]. School-based prevention programs underscored the importance of the intervention by reducing psychological, physical and sexual dating violence [16-19], this was further confirmed in reviews which reported multi component programs reduce the assaults and incidence of domestic violence [7,20,21]. Considering these evidence, preconception counseling should be made available especially in schools, community centers, and adolescent health centers. These facilities can provide a space where sexually active young men and women know the risks of teen pregnancy, understand the use of condoms as well as long-acting methods of contraception. Sexual education appears to be more effective if it is integrated with broader life skills and education programs, including vocational abilities, effective communication, responsible decision making, proactive approach to personal health care and an increased awareness of health related issues. Since academic failure and teenage pregnancies are seen to be so closely interlinked, a special emphasis should be made to ensure that teenage girls complete a basic secondary education which will include educating young minds on preventing teenage pregnancies. The purpose of education is to develop the knowledge to observe, understand, reason and make rational judgment about the known realities of the world for optimum survival. Teens that have dropped out of high school are more likely to become pregnant, and their children are more likely to be deficient in terms of child development milestones such as language, communication, and cognition [22]. This has long-term implications for them as individuals, their families and communities [23]. Analysing the key issues in a community may help to identify what or who are the major contributors; for example service and health care providers may not be offering adequate counselling, sexual health information or contraceptive methods, or school systems may be deficient in providing a comprehensive sexual health class or may have failed to provide a safe environment where teens may discuss their problems. In short, adolescent sexual and reproductive health programs should include counselling, reproductive planning and contraception, and screening and management of sexually transmitted infections (STIs). On the other hand, girls in marginalized areas can be provided with preconception counseling in community settings by community health workers and outreach workers [7].

A teenager's biggest asset is knowledge. Education is the gradient process of acquiring knowledge. Knowledge gives a teenager the tools for rational reasoning and judgment. Knowledge helps a teen make better choices. Knowledge gives you options. The more knowledge a teen has, the more opportunities a teenager will have for making money. It is, therefore, important to underscore the benefits of completing secondary education among adolescents.

#### 2. Family planning and nutrition counselling

Nutrition plays a paramount role in supporting the future pregnancy. Nutritional counselling of women prior to conception receives less importance compared to nutritional counselling of pregnant women; however if family planning and reproductive health services took a more integrated approach and included nutritional counselling – this could improve coverage. Preconception counselling will allow women at higher risks of malnutrition, such as older women (older than 35), teenage girls with restricted diets, or malnourished women with vitamin deficiencies, to be highlighted before conception so that appropriate action may be taken. Adolescents typically consume a diet that does not satisfy the required intake of iron, folate, or vitamin B6 and have high chances of being anaemic. Thus an integrated approach should be applied wherein couples who come in for family planning services may be targeted for nutrition counselling since they are at a preconception stage where they are looking to plan a future family and thus would want to adopt the best practices and habits to do so. In low and middle income countries (LMICs) and in marginalized parts of high income countries (HICs) where food insecurity is an issue, community programs to grow food or buy it collectively should be incorporated into nutritional programs.

##### Pre-conception counselling

There is evidence that preconception counselling changes risk behaviours and results in improved maternal and neonatal outcomes [24-43]. This includes risk assessment and counselling of women over the age of 35 on the increased risks associated with advanced maternal age [44-83], as well as genetic counselling of couples in a consanguineous marriage to ensure a healthy fetal outcome [84-88]. A closer look is taken into past obstetrical and gynecological history, past medical and drug history, chronic illnesses, family history of genetic disease and psychosocial history to rule out any potential complications of pregnancy. However, further randomized controlled trials are needed to show how preconception care affects maternal, newborn and child health (MNCH) outcomes, and to delineate where and by whom such care should be provided, how long before conception such care should begin, and which interventions are most successful.

##### Periconceptional folic acid supplementation

Folic acid supplementation is known to reduce the risk of neural tube defects (NTDs) in the newborn [89-93]. Many women are still unaware of how much their nutritional status impacts their pregnancy outcomes, and improving women’s nutritional behaviours should therefore begin during their earlier reproductive years. Women wishing to conceive should take 400ug of folic acid three months prior to pregnancy [94]. Women who have previously given birth to an infant with a NTD require higher levels of folic acid supplementation (800μg). Providers should assess women’s dietary habits and discuss the importance of micronutrients as part of routine preconception counseling. Other nutrition-specific interventions (iron, calcium, balanced protein energy supplementation etc.) have only been studied in pregnant women, or if they have been studied during the preconception period the outcomes are limited to changes in biochemical markers while pregnancy and birth outcomes were not assessed [95-100].

##### Family planning

One of the reasons for the slow progress towards millennium development goal (MDG)-5 is the inadequate delivery of family planning interventions, resulting in unintended pregnancies, subsequent abortions, and a subsequent rise in maternal mortality**.** A recent report demonstrated a high unmet need for family planning in countries like Mali and Chad with the lowest contraceptive prevalence rates [101]. The evidence showed an impact of long (>60 months) and short inter-pregnancy intervals (IPIs) (<6 months) on preterm births, low birth weight and small for gestational age babies [102-122]. The evidence suggests that counselling can help women understand the possible risks to themselves and their children of very short and very long IPIs, and the risks of having an unintended pregnancy and unsafe abortion [123]. Women should be advised to wait 18-24 months after pregnancies ending in a live birth, and at least 6 months after an abortion, before conceiving again, and should be provided with appropriate contraceptive counselling.

#### 3. Nutritional optimization and obesity prevention and management

Evidence has showed that pre-pregnancy underweight and overweight are risk factors for an array of composite adverse pregnancy and neonatal outcomes [63,124-155]. It is suggested that pre-pregnancy body mass index (BMI) should be maintained within the normal range of 18.5–24.9 kg/m^2^ and controlled via diet and exercise modifications (Refer to Paper 3[8]). The interventions for healthy diet and exercise should be encouraged from late childhood and early adolescent years to be effective [156-166]. Routine preconception care with regards to weight should include calculating BMI for women of reproductive age, increasing awareness regarding the risks associated with being overweight or underweight, and helping women develop individualized dietary plans including consumption of a variety of healthy foods in appropriate amounts, and dietary supplements (especially a multivitamin containing 400 μg of folic acid, calcium and vitamin D, and iron). Balanced protein energy supplementation and appropriate micronutrient supplementation can reduce the risk of outcomes related with pre-pregnancy underweight, especially in LMICs. All women should be encouraged to get their risks assessed for cardiovascular health with regards to weight bearing and offered appropriate lifestyle modifications. Women with high BMI’s should be counselled to understand the risks of infertility that are a part of increased weight gain. A focus on screening, treating and preventing sexually transmitted infections (STIs) in the preconception period is vital, as well as educating parents about the risk of vertical transmission of such infections to the child.

#### 4. Multicomponent youth development programs including prevention of sexually transmitted infections

Multicomponent youth development programs which encompass social, family, peer, community, educational and health disciplines are deigned to meet youth developmental needs and help them become healthy, happy and productive adults. Youth and particularly older children should be encouraged to take part in these programs. A proper and effective program will also help reduce problems such as substance abuse and teenage pregnancies [167-169]. Mentoring will be an essential component of these programs where developing youth are supported and encouraged to develop competencies, to take on leadership responsibilities, and to integrate into positive peer groups. By focusing on health and self-confidence issues, guidelines on how to eat healthy and reach desired BMI, as well as sexual health issues such as practicing safe sex and when and how to deal with STIs may be included. Programs that deal with alcohol or substance abuse as well as smoking cessation programs [29] can also be packaged into these multicomponent youth development programs to be highly effective [11].

##### Sexually Transmitted Infections

The review of evidence highlighted the wide gaps in current knowledge regarding the effects of treatment of STIs in the preconception period on combined maternal and neonatal outcomes [170-172]. Evidence from intervention that delivered behavioural counselling showed reduction in re-infection and new incidence of STI , thus this can reduce transmission of infection to the newborn, as well as improve the health of the woman during pregnancy and in the first year after birth [173-175]. Preventive care can be implemented with the application of preconception care by raising awareness on STIs and their symptoms and prevention, cervical screening, and PAP smears. All women of childbearing age should undergo testing for STIs. It is, however, warranted that more studies be conducted regarding STI treatment and counselling in the preconception period on maternal behaviours and future maternal and neonatal outcomes [9].

##### HIV/AIDS prevention strategies

All men and women should be encouraged to find out their HIV status before starting a family which can be a part of preconception counseling; if women test positive, they should be educated in detail of the risks of vertical transmission to the child and the risks for increased mortality and morbidity that is associated with this disease. Various strategies (e.g. prophylactic treatment of reproductive age women with antiretrovirals to prevent HIV transmission to her partner and newborn; condom use to prevent HIV transmission; counseling and voluntary testing to lower the risk of transmission through unprotected intercourse; and treatment of STIs for risk reduction) for preventing HIV/AIDS were reviewed [167,173,176-228]. Preconception care for women living with HIV/AIDS or HIV-positive partners is recommended. This should include effective and appropriate contraception to reduce the chance of unintended pregnancy, psychological and emotional support to encourage disclosure of sero-status to partner, education regarding the risks of perinatal transmission and strategies for prevention and screening, and treatment for STIs in partners [9].

#### 5. Screening and management of chronic diseases including mental health

The evidence highlights the importance of women’s health status including the presence of chronic diseases, current infections or syndromes, and promotes general screening and management for other chronic health related matters [229-264]. Another major component of health, which is mental health, is also highlighted as a package to reduce depression or anxiety disorders which may have adverse effects on the child’s health [265,266]. On par with that, domestic violence will also have a negative impact on emotional health and mental wellbeing such as posttraumatic stress disorder or psychiatric issues with abuse or rape [267-315]. This package serves to highlight the importance of empowering women to take control of their own health issues, to report physical or sexual harassment and abuse, and to be proactive in matters concerning their own health by screening and testing for chronic infections before planning a pregnancy [10,316-328].

All women who are chronic carriers of a disease such as hepatitis B should be counselled to receive vaccination if not done previously and instructed on how to prevent vertical transmission to future child or horizontal transmission to close contacts. Women with chronic health problems such as hypertension or hyper/hypothyroidism should be counselled about the risks associated with their disease during pregnancy, and the necessity to change medication regimens while pregnant or conceiving to optimize hormonal levels and to prevent any harm to the foetus. Management and counselling of diabetic women during the preconception period is more beneficial than during pregnancy [329,330]. Preconception care for women with pre-gestational diabetes should include education about the importance of strict glycaemic controls (with an HbA1C level of less than 6-7%) to prevent congenital anomalies; teaching self-management skills with pre-set monitoring targets; counselling on the effect of poor glycaemic control on maternal complications and fetal complications; counselling about diet (as per protocol for diabetes); and healthy physical activity for weight management [10]. Testing to detect pre-diabetes or type-2 diabetes should be a priority for high-risk women who are obese or overweight or those who have a strong family history of diabetes.

##### Maternal Mental health

Mental health issues in mothers often remain undiagnosed and their management often overlooked. Evidence suggests linkages between poor adolescent mental health and poor pregnancy and postpartum outcomes [10,331-334]. Preconception care for psychiatric conditions is recommended; this should include screening of women in their childbearing years for mood disorders and identifying those at risk; counselling women with pre-existing depression and anxiety disorders about the potential risks of untreated illness and its associated negative health outcomes; and informing potential mothers about the risks and benefits of various treatment options prior to conception and during pregnancy. Women of reproductive age must be counselled that relapse might be a complication during pregnancy for bipolar disorder or schizophrenia patients since they will be discontinuing treatment. A relapse prevention and treatment strategy should be drawn out as part of a pre conception plan.

##### Prevention of domestic violence

Domestic violence in females is a major contributor to ill health, particularly to their reproductive and sexual health, and it is also a violation of human rights which is mostly brought about due to gender inequality. A growing problem in both LMICs and HICs, physical abuse leads to negative outcomes such as injuries, trauma, unwanted pregnancies, and emotional disturbances [270,281,288,295,296,335]. In areas or societies where domestic violence is a common practice, females might not have the autonomy of making decisions related to their reproductive health which leads to a higher risk of contracting sexually transmitted infections as well as unwanted pregnancies, or multiple pregnancies without adequate time to recover. Women who are subject to regular acts of violence may be victims of such abuse even during pregnancy, which has deleterious effects on the foetus. General support groups may be able to reduce incidences of domestic violence or at least raise awareness among such females, and build a system where such women can feel safe reporting such incidences [10].

## Opportunities for delivery

Interventions that are implemented in various settings particularly in low-income countries face the challenge of a lack of standardization across the line of service delivery, community outreach, and organizational policies. Each setting has different requirements, which might bring about healthcare inequities, but can be substantially reduced by integrating intervention packages into existing local health programs. To ensure sustainability and to be as efficient as possible at meeting health care outcomes, the measure of integration with existing health care systems and within other delivery platform is vital; not only will it help in overcoming barriers to service delivery and accessibility, but output delivery can be amplified. As a result, existing systems are strengthened and improved further, sparking positive development in the form of viable healthcare networks in such communities. To ensure that the packages of care that have been a product of much research and knowledge are translated into action, the following opportunities for delivery and integrating services within the broader platform can provide a means to do so.

### a) Delivery within the education system

#### School health and reproductive health education programs

Information and services must be made available to adolescents to educate them on their sexuality and protect themselves from unwanted pregnancies, STIs, or risks of infertility. School-wide accepted sexed programs have shown to delay the onset of sexual activity and increase safer sexual practices by those that are already sexually active. All females should be counselled through regular school health programs about eating disorders, such as anorexia or bulimia and the risks to fertility and future pregnancies it might have. The reproductive and sexual health program should be integrated as a vital component of the school curricula and must be tested upon to emphasize its importance. Adolescents should be guided as to how to make responsible decisions concerning their sexual lives, as well as how to practice safe sex, and how to prevent unwanted pregnancies.

### b) Delivery within health system

#### Primary-level health workers (e.g. community health workers)

The primary level health workers are the backbone to the whole establishment of primary health care. By delivering health services to the doorsteps of the community as in the case of community health workers (CHWs), concepts such as family planning, vaccinations or immunizations, proper growth monitoring, nutrition for neonates and control of common diseases can be implemented. CHWs can be trained to offer guidance and increase awareness regarding STIs, to assess maternal health and nutrition status in order to improve postpartum outcomes. They can also provide information about all forms of contraceptives, their benefits and efficacy and also provide information on emergency contraception to the female population of child bearing age in a community. Folic acid, iron and micronutrient supplementations may also be distributed in this way along with a basic orientation of the importance of such supplements to improve maternal and child health. Health education that is a vital part of preconception care and health promotion, but which may be impractical to distribute effectively in most clinical settings, especially in busy health clinics with large patient to doctor ratios can utilize CHWs to increase awareness levels.

#### Pre-marital counseling and screening

Pre-marital counselling and screening allows couples to test for the presence of infectious diseases such as HIV/AIDS, Hepatitis B and C, syphilis or for genetic diseases such as beta-thalassemia or sickle cell haemoglobinopathies in both male and female to ensure proper care is taken before a pregnancy is planned. Such services should be available at all clinics/outreach clinics and doctors should make patients aware that this is a necessary and important step to take before starting a family. Regular screening should be conducted in high risk families by testing them and having them sit down with a geneticist and receive counselling on the best course of action. This would greatly reduce the occurrence of hereditary disorders.

#### Expanded post-natal care

During the first days to weeks after birth, the postnatal period is the ideal time to deliver interventions to improve the health and survival of both the newborn and the mother, a facility that a majority of mothers currently avail. However, an efficient strategy would be to expand post-natal care to include preconception care as well to optimize chances of a healthy conception, pregnancy and delivery. Programs should also aim to provide earlier and more integrated care as a majority of pregnancy complications may be avoided with proper screening, personal history, nutritional counseling, and genetic counseling so parents can be better equipped to handle a pregnancy and subsequent birth.

#### Take advantage of other health visits and missed opportunities

For most patients coming in for regular check-ups to a general physician in walk-in clinics, emergency department and sports medicine clinics must take an accurate and detailed history complete with relevant tests and refer to a specialist when needed. The doctor can also offer basic care and guidance regarding reproductive health to patients to improve maternal, neonatal and child health.

#### Add preconception-related items to provider checklists

Integrate essential components of nutrition such as folic acid, iron, zinc, and micronutrient supplementation alongside programs that have a high coverage such as vaccination or immunization programs which could target both pregnant women as well as neonates.

### c) Other platforms

#### Community support groups

Community-based programs that provide pre-conception services will cater directly to the specific needs of the particular community. Instead of a larger scale system of preconception care, support groups will be able to distribute care into smaller communities, villages, and rural areas. Members of support groups are usually facing similar issues and can benefit greatly from the shared experiences, advice and health support of the other participants. Key issues of the community such as a high rate of teenage pregnancies or poor family planning services can also be targeted and interventions can then be tailored and implemented accordingly. For example, through support group sessions, teenage girls can be taught confidence and empowerment skills to contest early or forceful marriages; they can also be taught some sort of self defence mechanism if rape and unwanted sexual advances are an issue.

#### Mass media campaigns/ Social marketing

Modern marketing techniques and social communications have been successfully used in many regions to promote the importance of reproductive health. Many of the interventions can be delivered through health facilities but to reach a larger audience mass mobile text messages or radio announcements may also be used where applicable. Mass campaigns regarding immunization programs in a locality or on the importance of family planning and availability of family planning clinics in different areas may be advertised on TV channels, radio programs or through text messages. Mass media campaigns for TV, radio and print media may be utilized to promote safer sexual practices, especially using condoms to prevent STIs. Local celebrities or personas may be used to promote a healthy image of utilizing pre-conception care at local health facilities. A challenge faced by many at-risk young adults in developing countries is the fact that most of them are illiterate, or have poor exposure to educational programs – mass media may be a very useful tool for effectively transmitting basic health messages to such a population. It can also be an instrument for changing behavioural stereotypes, pre-formed attitudes, myths and misconceptions regarding reproductive health.

#### Workplace programs or referrals

Educational workshops may be conducted that are mandatory in workplace venues where men and women both need to attend sessions on sexual health/STI’s, smoking cessation, and other health-related topics. By providing access to sexual rehabilitation programs for employees and their spouses, which would include coping mechanisms for infertility, menopause, late pregnancies, miscarriages, or pregnancy complications, a significant reduction in risky sexual behaviors and practices as well as distress resulting from infertility or other issues may be achieved. A high prevalence of sexual problems with age may be secondary to other medical conditions which must be investigated; when left untreated, these problems will lead to a decreased quality of life, depression, peer and spouse conflicts.

#### Food fortification

Food fortification is the practice of adding micronutrients to processed foods for an increase in the micronutrient status of a population and a decrease in deficiencies and related programs. A very cost-effective program, the addition of folic acid to enrich flour, rice and pasta takes advantage of existing technologies and local distribution networks.

#### Support groups for individuals with a particular risk

Support groups for high-risk couples and women, including those who have experienced a previous poor outcome. Such programs provide outreach, case management, risk reduction, support, prenatal/preconception care, health education and community development to high-risk women and couples. Such women and couples who have had a previous miscarriage, delivery of a low birth weight (LBW) infant, lack of family planning, repeated STI’s, early pregnancies, substance abuse, and lack of access to reproductive health care would be the target group for maximum benefit. The individual’s case would be assessed and a goal plan drawn out involving the patient at every step; from here on, the support group staff ensures compliance with services and keeps updated with the achievement of personal goals.

## Discussion

This paper has highlighted the inextricable link between maternal and newborn health by providing evidence of the effect of interventions delivered during the preconception period on maternal and neonatal outcomes. Only a few systematic efforts have identified potential synergies between key reproductive health interventions on maternal and perinatal outcomes. This paper identified papers that underscored these synergies and illustrated a number of important interventions that should be delivered during the preconception period. Most of the interventions reviewed will require additional high quality evidence before recommendation for their implementation in programs.

Considering the interdependent relationship between maternal and neonatal health, approaching interventions that have a direct effect on MNCH services has great potential for accelerating progress towards MDG 4 and 5. The integration of these interventions needs to span not only in time period (pregnancy, childbirth, and the postnatal period) but also levels of care (household, community, and health facilities). The delivery of these interventions can have profound translational and intergenerational impacts with important implications for the long-term well-being of both mother and newborn. This can also promote greater efficiency by maximizing synergies and avoiding duplication of services that are less efficient if delivered individually. Therefore, these interventions should be integrated in health policies and programs. Such potential integration of strategies would not only help improve maternal and newborn outcomes but would also save scarce resources. This approach is critical to achieving MDGs 4 and 5 and making sure that we progress beyond saving lives to also improving morbidities and other developmental outcomes.

At the present time, preconception care is not implemented at a global level, and is not a widespread concept in LMICs as of yet. These packages feature intervention packages, opportunities for delivery and highlight the implementation of intervention packages via existing health care and public health programs. An emphasis is placed on the importance of expanding already existing programs such as antenatal care to include preconception care which would be an effective strategy in low-income countries and would capture a wide audience. To implement on a community level, an understanding and awareness should be developed for all stakeholders so that healthcare providers as well as higher policy makers may reach a shared consensus. A shared goal and a quantifiable and easily measured objective must be drawn out such as a reduction in maternal and infant mortality by a predetermined date in time or a significant decrease in maternal and neonatal infections for example. Organizations who propose to forward the preconception care packages and health care providers, as well as primary level health care workers such as community health workers should develop an agenda for goals, objectives, action plans, and evaluation strategies. Those organizations that have prior experience in delivering preconception care could be invited to give an expert opinion in what approaches are effective or what errors may be avoided.

Since malnutrition and vitamin and nutrient deficiencies contribute globally to a huge burden of disease processes, from LMICs to HICs, packages focused on supplementation strategies such as folic acid or micronutrient supplementation, and fortification of foods should be implemented on a national level with strict guidelines and limitations. Iodine, iron, vitamin A, and zinc were identified being among the world’s most serious health risk factors leading not only to clinical manifestations such as anaemia or metabolism disorders but also contributing to delayed motor and cognitive development. Food fortification, when implemented on a national level, with effective policies to govern safe practices, will be able to target a large segment of the population. In countries where vitamin D levels has been tested and known to be deficient in over 90% of the population, vitamin D fortified milk should be made available at all places. Where the level of deficiencies are too high such as populations where anaemia rates are endemic, then accessibility of the fortified foods to poorer settlements must be taken into concern or the presence of existing infections must be evaluated since it will raise the metabolic requirement for the micronutrient.

Multi-component youth development programs, sexual education programs, and community support groups as packages should be targeted to empower women and girls to take control of their own health especially reproductive health, to make responsible and autonomous decisions regarding their sexual life, to prevent or report domestic violence, and to prevent sexually transmitted infections by being firm on the use of contraceptives. This can be implemented at the community level with regards to specific issues of each community such as a high rate of rape cases may be countered with increased awareness and availability of emergency contraception and promotion of self –defence.

Other packages such as nutritional counselling, genetic counselling, and pre-marital screening should be targeted to involve both male and females as a couple and the focus should be on the health and wellbeing of the future family including parents and the child. Other packages such as family planning or contraceptive methods should take a multi-centred approach with delivery through various forums such as awareness through workshops and community support groups, provision of subsidized condoms in a poor community, advocating the benefits and warning of the risks of infections through health care facilities.

For implementation in LMICs at a community and facility level, cultural and social diversities and customs must be taken into account. Many regions and areas have deeply embedded social norms and traditions that govern their people’s beliefs and actions; an example would be of religious barriers to contraceptive methods practiced in a majority of rural or low income communities especially in South East Asia. This would provide a significant barrier to preconception care packages such as family planning packages, which rely on effective contraceptive practices, or packages that advocate the use of condoms to prevent STIs. CHWs can be directed to raise awareness levels in such communities while delivering packages as doorsteps as well as conducting community level workshops.

Once a shared consensus of the aims and objectives are drawn out, all stakeholders must be engaged from the community to the national level; those organizations such as non-profit organizations which are working on a sole purpose such as reducing HIV/AIDS prevalence may be incorporated into this wider program and be used to reach a larger target audience. Rehabilitation centres for substance abuse, smoking cessation clinics, and other outlets may be utilized as well to bring about a combined preconception package to a much larger segment of the population. Areas where these packages may not be reaching such as prisons or homeless shelters for example must have a specified implementation package as well to ensure as large a distribution of preconception care as possible.

## Conclusion

Since reducing maternal and child mortality and morbidity rates are of utmost and urgent concern in LMICs and more specifically in certain regions in these countries, then preconception care can address such populations with the appropriate packages of care. To achieve that significant reduction in maternal and child mortality rates, existing care that includes pregnancy care, as well as antenatal care should be extended to include preconception care as well as take into account adolescents, women of child-bearing age, and all high risk women. A focus on prevention of infections or deleterious habits, which will eventually become complications during pregnancy and a primary focus on improving reproductive health, will be the cornerstones of the implementations of these packages.

## Competing interests

We do not have any financial or non-financial competing interests for this review.

## Peer review

Peer review files are included in additional file 1.

## Supplementary Material

Additional file 1 Peer review reports Click here for file

## Declarations

This article has been published as part of *Reproductive Health* Volume 11 Supplement 2, 2014: Preconception interventions. The full contents of the supplement are available online at http://www.reproductive-health-journal.com/supplements/11/S3. Publication charges for this collection were funded by the Partnership for Maternal, Newborn & Child Health (PMNCH).
