# Supplementary material for: Preconception care: delivery strategies and packages for care
Source: Reprod Health. 2014 Sep 26;11(Suppl 3):S7. doi: 10.1186/1742-4755-11-S3-S7 (PMC4196568; doi:10.1186/1742-4755-11-S3-S7)
Supplement: Additional file 1 — Peer review reports [file 1742-4755-11-S3-S7-S1.pdf]

Referee's comments to the authors– this sheet WILL be seen by the author(s) and published with the article

Title	**Preconception care: delivery strategies and packages for care**
Author(s)	Zohra S Lassi, Sohni V Dean, Ayesha M Imam, Zulfiqar A Bhutta
Referee's name	Miriam Kaufman

When assessing the work, please consider the following points, where applicable:

1. Is the question posed by the authors new and well defined?
2. Are the methods appropriate and well described, and are sufficient details provided to replicate the work?
3. Are the data sound and well controlled?
4. Does the manuscript adhere to the relevant standards for reporting and data deposition?
5. Are the discussion and conclusions well balanced and adequately supported by the data?
6. Do the title and abstract accurately convey what has been found?
7. Is the writing acceptable?

Please make your report as constructive and detailed as possible in your comments so that authors have the opportunity to overcome any serious deficiencies that you find and please also divide your comments into the following categories:

- Major Compulsory Revisions (which the author must respond to before a decision on publication can be reached)
- Minor Essential Revisions (such as missing labels on figures, or the wrong use of a term, which the author can be trusted to correct)
- Discretionary Revisions (which are recommendations for improvement but which the author can choose to ignore)

Where possible please supply references to substantiate your comments.

When referring to the manuscript please provide specific page and paragraph citations where appropriate.

General comments: The term "pre-conception care" is somewhat problematic. It seems to assume that young people have the single goal of creating progeny and everything they do before procreating is preparation for the event. All of the packages of care that are discussed in this review are terrific and almost all would contribute to the overall well-being of the entire population, whether or not they end up having children. It would really be better to address the issue of good adolescent health care and how it has a positive impact on growth and development, adult health, and pregnancy and pregnancy outcomes, rather than looking through the other end of the telescope.
I was very happy to see the inclusion of attention to issues of mental health, as they are a growing concern and a real issue for young parents. Similarly, young mothers, particularly those whose children have older fathers, are at risk for abuse and the attention paid to it in this paper is an important start.

Some thoughts on some of the individual packages:
1. Pre-marital counselling does not really apply to the young mothers and fathers who are not married. These interventions should all be able to embrace the most marginalized of adolescents, and these include teens who are not married. There are countries where the vast majority of mothers are married and in some of these there is mandatory counselling before writing and signing a marriage contract. There could be a much expanded role within this counselling as outline in the package.

(continue on the next sheet)

2. Tiny numbers of adolescents attend doctors, nurses or other health care workers for regular check-ups. They are more likely to go for sports injuries, acute infections and contraception. Rather than making recommendations for regular check-ups, it might be better to make recommendations for quick, preventative services that can be offered by walk-in clinics, emergency departments or sports medicine clinics.

Revisions:
The authors refer to the paper in a number of places as a review, but there are only three references. Are they reviewing packages that they have designed and tested? If so, this should be made clear. Otherwise, the references for these interventions should be included. If some of the packages have been implemented by not tested, we should be told this and know where they have been implemented.

Title	**Preconception care: delivery strategies and packages for care**
Author(s)	Zohra S Lassi, Sohni V Dean, Ayesha M Imam, Zulfiqar A Bhutta
Referee's name	George Patton

The paper deals with an important emerging question of the scope for interventions that affect embryonic and fetal development prior to the institution of normal antenatal care. These are interventions prior conception and continuing into the time before pregnancy is typically first recognised.

The paper is generally clear in what it communicates but at times is repetitive, could be better organised and probably requires a great level of critical detail if it is to be stand-alone. The approach is not to go to a level of detail that would be needed for formulating a standard clinical intervention package. Rather it is to outline broad areas for clinical consideration in women prior to conception.

The main difficulty that I had was that I was reviewing this paper in isolation from the rest of the supplement. I assume that details of the review appear elsewhere in the supplement which may be fine for the reader but for review left me unclear about what methods had been pursued. For that reason this review is necessarily limited in scope.

The authors introduce comments about birth space and teenage pregnancy that I would generally agree with but it is striking that few if any comments are referenced. The lack of referencing continues through the document. Again that may be fine given that this appears to be a summary paper for the supplement but again makes it difficult for review.

The framework for preconception intervention is clear and outlines the scope for intervention. One potential limitation is that intervention for some maternal risks cut across the different levels. Both weight management and tobacco use fall into that category. That is true of the provision of effective contraception to teenage girls where it is not just supply factors and skills of professionals that are barriers, but also community attitudes, particularly towards sexually active unmarried young women.

The interventions in school are at odds with the title. The summary addressing knowledge and counselling around sex but the title addresses completion of secondary education. There should be a greater alignment of the content with the heading.

Tying nutritional counselling into family planning appears to make sense. The main nutritional intervention mentioned in this section is folate. I was a little surprised as I might have thought that other nutrients may also be important in different contexts e.g. iron deficiency. There is generally a failure to summarise what are the major nutritional deficiencies that might be in focus for preconception intervention. I did wonder if this should be combined with elements around underweight and overweight in the next section. Again this may have been covered elsewhere in the supplement but if this paper is a summary overview some indication of the scope of nutritional intervention should still be provided here.

The following section 3 covers a mixture of interventions ranging from the nutritional to psychosocial. The tone of the section is prescriptive and with little mention of timelines. I suspect that we do not know how long it takes to reverse metabolic risks associated with obesity to reduce pregnancy risks for mothers and their offspring. But if this is so, it would be useful for the authors to let the reader know. So too, those aspects of positive youth development often begin to emerge in the late childhood years and intervention probably needs to begin then to be effective. Again there is a lack of reference to intervention studies. Again I suspect there is a very limited literature but it would be good to remind the reader of this.

The mention of mental health in section 4 is welcome as are the links to violence and abuse. I did wonder if the organisation of chronic physical illness with mental health made sense. I think that it may have made sense to separate these elements and remind the reader how commonly they might expect to find these problems in their clinical care.

Opportunities for delivery are very important for consideration and the authors have flagged a number of strategies and settings. As with the other sections there are no details provided of the expected range of coverage through these different strategies. Again this probably reflects the structure of the series of papers. The main problem is that without this more detailed information the reader is unable to usefully interpret the information. For example, sexual education in schools has a pretty mixed record with definite evidence that some but not all approaches can make a modest difference in the short term to sexual health risks. This is not clear at the bottom of page 7. And as far as I am aware there have never been any demonstration that counselling for eating disorders has ever had any effects on any health outcome.

This section on opportunities for delivery might be better organised around the conceptual framework and levels of delivery. It is currently difficult for the reader to follow and it jumps from clinical to community settings without any clear order.

The conclusion is not particularly useful and comes across as a further repetition of what has come before and I don't think it adds much. The point about community engagement is new but my sense is that this should appear earlier on and could do so if the section on 'opportunities' was re-organised.

Supplement Editor's comments

It is important that all the articles in the supplement can be read and understood independently of the other papers in the supplement.

Referee's comments to the authors– this sheet WILL be seen by the author(s) and published with the article

Title	**Preconception care: delivery strategies and packages for care**
Author(s)	Zohra S Lassi, Sohni V Dean, Ayesha M Imam, Zulfiqar A Bhutta
Referee's name	Miriam Kaufman

When assessing the work, please consider the following points, where applicable:

1. Is the question posed by the authors new and well defined?
2. Are the methods appropriate and well described, and are sufficient details provided to replicate the work?
3. Are the data sound and well controlled?
4. Does the manuscript adhere to the relevant standards for reporting and data deposition?
5. Are the discussion and conclusions well balanced and adequately supported by the data?
6. Do the title and abstract accurately convey what has been found?
7. Is the writing acceptable?

Please make your report as constructive and detailed as possible in your comments so that authors have the opportunity to overcome any serious deficiencies that you find and please also divide your comments into the following categories:

- Major Compulsory Revisions (which the author must respond to before a decision on publication can be reached)
- Minor Essential Revisions (such as missing labels on figures, or the wrong use of a term, which the author can be trusted to correct)
- Discretionary Revisions (which are recommendations for improvement but which the author can choose to ignore)

Where possible please supply references to substantiate your comments.

When referring to the manuscript please provide specific page and paragraph citations where appropriate.

General comments: The term "pre-conception care" is somewhat problematic. It seems to assume that young people have the single goal of creating progeny and everything they do before procreating is preparation for the event. All of the packages of care that are discussed in this review are terrific and almost all would contribute to the overall well-being of the entire population, whether or not they end up having children. It would really be better to address the issue of good adolescent health care and how it has a positive impact on growth and development, adult health, and pregnancy and pregnancy outcomes, rather than looking through the other end of the telescope.

This is the definition by World Health Organization and the idea is to provide a set of biomedical, behavioral and social health interventions to women and couples before they plan pregnancy. From this pool some may end up having children and some may not but these interventions will prepare them for healthy pregnancy and child health outcomes.

I was very happy to see the inclusion of attention to issues of mental health, as they are a growing concern and a real issue for young parents. Similarly, young mothers, particularly those whose children have older fathers, are at risk for abuse and the attention paid to it in this paper is an important start.

Thanks. We included this with the same reason.

Some thoughts on some of the individual packages:

1. **Pre-marital counselling does not really apply to the young mothers and fathers who are not married. These interventions should all be able to embrace the most marginalized of adolescents, and these include teens who are not married. There are countries where the vast majority of mothers are married and in some of these there is mandatory counselling before writing and signing a marriage contract. There could be a much expanded role within this counselling as outline in the package.**

   This section has been covered at two places. One under the heading of "Completion of secondary education for adolescent girls and prevention of teenage pregnancy" and the second under the heading of Nutritional counselling and family planning which has a subpart of preconception counselling". Also added few lines "On the other hand, girls in marginalized areas can be provided with preconception counseling at community level by community health workers and outreach workers."

2. It is great to assess young women's dietary intake and to counsel them about it, but food insecurity is a huge issue throughout the world. It is not just an issue for the underweight teen, but more and more, overweight teens are undernourished in all but calories. Community programs to grow food or buy it collectively should be incorporated into nutritional programs.

We understand this is important but the recommendations are based on evidence we gathered in the review. We have now added a reference to the paper where these findings have been discussed. Also added the important point suggested.

Major compulsory revisions:

Minor essential revisions:

2. **Tiny numbers of adolescents attend doctors, nurses or other health care workers for regular check-ups. They are more likely to go for sports injuries, acute infections and contraception. Rather than making recommendations for regular check-ups, it might be better to make recommendations for quick, preventative services that can be offered by walk-in clinics, emergency departments or sports medicine clinics.**

We have incorporated this comment and added into the section as "For most patients coming in for regular check-ups to a general physician in walk-in clinics, emergency department and sports medicine clinics must take an accurate and detailed history complete with relevant tests and refer to a specialist when needed."

Revisions:
The authors refer to the paper in a number of places as a review, but there are only three references. Are they reviewing packages that they have designed and tested? If so, this should be made clear. Otherwise, the references for these interventions should be included. If some of the packages have been implemented by not tested, we should be told this and know where they have been implemented.

We have now provided references to the papers where that section has been described in detail. Here we are suggesting packages based on what we have reviewed in the papers (and provided references to).

Title	**Preconception care: delivery strategies and packages for care**
Author(s)	Zohra S Lassi, Sohni V Dean, Ayesha M Imam, Zulfiqar A Bhutta
Referee's name	George Patton

The paper deals with an important emerging question of the scope for interventions that affect embryonic and fetal development prior to the institution of normal antenatal care. These are interventions prior conception and continuing into the time before pregnancy is typically first recognised.

The paper is generally clear in what it communicates but at times is repetitive, could be better organised and probably requires a great level of critical detail if it is to be stand-alone. The approach is not to go to a level of detail that would be needed for formulating a standard clinical intervention package. Rather it is to outline broad areas for clinical consideration in women prior to conception.

We tried reporting them in different ways prior to submission and then came up with this approach with which we all agreed to. Also, it was difficult and repetitive to provide detailed description in each paper, hence we have referenced the paper providing details

The main difficulty that I had was that I was reviewing this paper in isolation from the rest of the supplement. I assume that details of the review appear elsewhere in the supplement which may be fine for the reader but for review left me unclear about what methods had been pursued. For that reason this review is necessarily limited in scope.

We have now inserted the reference to the other papers in the supplement where the specific sections are described in detail.

The authors introduce comments about birth space and teenage pregnancy that I would generally agree with but it is striking that few if any comments are referenced. The lack of referencing continues through the document. Again that may be fine given that this appears to be a summary paper for the supplement but again makes it difficult for review.

Yes this is very true. All the references are mentioned in the main paper pertinent to topic. It would be difficult to adjust all those references here in this paper. We have therefore, inserted references to those descriptive reviews in this section.

The framework for preconception intervention is clear and outlines the scope for intervention. One potential limitation is that intervention for some maternal risks cut across the different levels. Both weight management and tobacco use fall into that category. That is true of the provision of effective contraception to teenage girls where it is not just supply factors and skills of professionals that are barriers, but also community attitudes, particularly towards sexually active unmarried young women.
The interventions in school are at odds with the title. The summary addressing knowledge and counselling around sex but the title addresses completion of secondary education. There should be a greater alignment of the content with the heading.
Tying nutritional counselling into family planning appears to make sense. The main nutritional intervention mentioned in this section is folate. I was a little surprised as I might have thought that other nutrients may also be important in different contexts e.g. iron deficiency. There is generally a failure to summarise what are the major nutritional deficiencies that might be in focus for preconception intervention. I did wonder if this should be combined with elements around underweight and overweight in the next section. Again this may have been covered elsewhere in the supplement but if this paper is a summary overview some indication of the scope of nutritional intervention should still be provided here.

In this paper we have only discussed the interventions reviewed in those individual sections which showed a significant impact on maternal, newborn and child health and hence enhanced focus on certain interventions. However, for further clarity, we have now inserted the references where these individual interventions are discussed in detail.
Re improving the section on sexual counseling and school education: We have now improved the section in the paper. We have added sentences to improve "The purpose of education is to develop the knowledge to observe, understand, reason and make rational judgment about the known realities of the world for optimum survival."

The following section 3 covers a mixture of interventions ranging from the nutritional to psychosocial. The tone of the section is prescriptive and with little mention of timelines. I suspect that we do not know how long it takes to reverse metabolic risks associated with obesity to reduce pregnancy risks for mothers and their offspring. But if this is so, it would be useful for the authors to let the reader know. So too, those aspects of positive youth development often begin to emerge in the late childhood

years and intervention probably needs to begin then to be effective. Again there is a lack of reference to intervention studies. Again I suspect there is a very limited literature but it would be good to remind the reader of this.

Again the references to the papers where those reviews have been mentioned in detail are given in the text. Also improved the section based on suggestion particularly for education and sexual intervention. “The interventions for healthy diet and exercise should be encouraged from late childhood and early adolescent years to be effective”.

The mention of mental health in section 4 is welcome as are the links to violence and abuse. I did wonder if the organisation of chronic physical illness with mental health made sense. I think that it may have made sense to separate these elements and remind the reader how commonly they might expect to find these problems in their clinical care.

We agree to the comment. We merged these sections because we wanted to suggest few packages and to enhance their deliverability as one. Mental health as whole has been highlighted in the section and we expect that readers will understand its importance as well.

Opportunities for delivery are very important for consideration and the authors have flagged a number of strategies and settings. As with the other sections there are no details provided of the expected range of coverage through these different strategies. Again this probably reflects the structure of the series of papers. The main problem is that without this more detailed information the reader is unable to usefully interpret the information. For example, sexual education in schools has a pretty mixed record with definite evidence that some but not all approaches can make a modest difference in the short term to sexual health risks. This is not clear at the bottom of page 7. And as far as I am aware there have never been any demonstration that counselling for eating disorders has ever had any effects on any health outcome.

We have now rearranged the sections under the opportunities heading. The arrangement is now starting from school program to community level programs to health facility programs. The citations to the references to the other reviews in the supplement will help readers to refer to the pertinent section which will allow more clarity.

This section on opportunities for delivery might be better organised around the conceptual framework and levels of delivery. It is currently difficult for the reader to follow and it jumps from clinical to community settings without any clear order.

We have now reordered them as per suggestion.

The conclusion is not particularly useful and comes across as a further repetition of what has come before and I don’t think it adds much. The point about community engagement is new but my sense is that this should appear earlier on and could do so if the section on ‘opportunities’ was re-organised.

Reorganized the section on opportunities. We have also revisited the conclusion section and have removed the repetitive parts unless where necessary.

Paper S7, reply to authors.

Abstract. The abstract should show the major results derived from this review. It is almost an abstract addressing the background and only a sentence: “This paper has highlighted packages of preconception interventions that can be bundled together and deliver to women through various underscored delivery mechanisms.” Please describe briefly the content of the packages and the delivery mechanisms in the abstract.

There is a scarcity of replies to reviewers included in the new version of the manuscript. Please, go again to each of the reviewers comments explaining where in the text you included such replies.

There are many recommendations including some of them as strong recommendations without a justification. There is a need to better support such recommendations by references or to soften the strength of it.

There is a scarcity of references in the manuscript, please provide them in the manuscript.

As the reviewers mentioned it is no easy to follow your manuscript without reading the other articles of the Supplement and I made you a recommendation that the articles could be read as independent articles without the need to go over the other articles of the Supplement.

Paper S7, reply to authors.

Abstract. The abstract should show the major results derived from this review. It is almost an abstract addressing the background and only a sentence: “This paper has highlighted packages of preconception interventions that can be bundled together and deliver to women through various underscored delivery mechanisms.” Please describe briefly the content of the packages and the delivery mechanisms in the abstract.

We have revised the abstract.

There is a scarcity of replies to reviewers included in the new version of the manuscript. Please, go again to each of the reviewers comments explaining where in the text you included such replies.

We have provided our revised answers to the comments in the sheet sent earlier.

There are many recommendations including some of them as strong recommendations without a justification. There is a need to better support such recommendations by references or to soften the strength of it.

Revisited the entire manuscript and provided references where required.

There is a scarcity of references in the manuscript, please provide them in the manuscript.

Added

As the reviewers mentioned it is no easy to follow your manuscript without reading the other articles of the Supplement and I made you a recommendation that the articles could be read as independent articles without the need to go over the other articles of the Supplement.

This paper can now be read independently as we have added a table of important preconception interventions reviewed in the paper and in the supplement.
